# Neurodevelopmental Disorders in Infants Treated with Anti-VEGF Agents versus Laser Photocoagulation or Cryotherapy for Retinopathy of Prematurity

**DOI:** 10.1016/j.xops.2026.101371

**Published:** 2026-08-17

**Authors:** Inès Ben Ghezala, Caroline Bonnet, Caroline Conese, Amir Hassine, Maxime Luu, Catherine Creuzot-Garcher, Marc Bardou

**Affiliations:** 1 Université Bourgogne Europe, CHU Dijon Bourgogne, Ophthalmology Department, Dijon, France; 2 Université Bourgogne Europe, CHU Dijon Bourgogne, Inserm, CIC 1432, Clinical Investigation Center, Clinical Epidemiology/Clinical Trials Unit, Dijon, France; 3 Observatoire Régional de la Santé Bourgogne-Franche-Comté, Dijon, France; 4 Eye and Nutrition Research Group, CSGA, UMR1324 INRAE, 6265 CNRS, Dijon, France

**Keywords:** Retinopathy of prematurity, Anti-VEGF, Laser photocoagulation, Drug safety, Neurodevelopmental disorders

## Abstract

**Objective:**

To compare the incidence of early neurodevelopmental disorders in very preterm infants treated with intravitreal anti-VEGF agents versus laser photocoagulation and/or cryotherapy for retinopathy of prematurity (ROP).

**Design:**

Nationwide population-based retrospective cohort study.

**Participants:**

All very preterm newborns (gestational age <32 weeks) born in France between 2015 and 2022 who received treatment for ROP with anti-VEGF agents, laser photocoagulation, or cryotherapy within the first 24 weeks of life.

**Methods:**

Using the French National Health Data System (Système National des Données de Santé), a medico-administrative database, we compared the incidence of early neurodevelopmental disorders between newborns treated with anti-VEGF agents and those treated with laser photocoagulation and/or cryotherapy, applying a propensity score‑weighted logistic regression model with inverse probability of treatment weighting.

**Main Outcome Measures:**

Incidence of early neurodevelopmental disorders within the first 6 years of life.

**Results:**

The number of infants treated exclusively with anti-VEGF agents increased by 300% from 2015 to 2022. The primary comparative analysis was conducted on 102 newborns born between 2015 and 2019, for whom mother–infant linkage was available in the Système National des Données de Santé: 59 treated with anti-VEGF agents and 43 with laser and/or cryotherapy. Early neurodevelopmental disorders within the first 6 years of life were not observed more frequently in infants treated with anti-VEGF agents than in those treated with laser and/or cryotherapy (71.4% vs. 58.1%; odds ratio, 1.4; 95% confidence interval, 0.8–2.4; *P* = 0.31).

**Conclusions:**

Using intravitreal anti-VEGF agents in very preterm newborns did not increase the risk of early neurodevelopmental disorders within the first 6 years of life. While a small excess risk cannot be formally excluded, these results might be reassuring for clinical practice, particularly given the substantial increase in the use of anti-VEGF agents for the treatment of ROP in recent years.

**Financial Disclosure(s):**

Proprietary or commercial disclosure may be found in the Footnotes and Disclosures at the end of this article.

Retinopathy of prematurity (ROP) is the leading cause of preventable childhood blindness worldwide,^1^ with an estimated prevalence ranging from 33% to 73% among preterm newborns with a gestational age below 27 to 28 weeks.^2^ In developed countries, the incidence of ROP tends to rise alongside increasing survival of very preterm infants and changes in oxygen therapy practices.^3^^,^^4^ Until recently, the current standard treatment involved laser photocoagulation to reduce retinal damage and the risk of poor visual acuity.^5^^,^^6^ In recent years, intravitreal injections (IVTs) of anti-VEGF agents have been proposed as an alternative treatment for ROP, after randomized controlled trials have demonstrated similar or superior efficacy compared with laser photocoagulation.^7^, ^8^, ^9^ Anti-VEGF IVTs offer several advantages over laser therapy: they can be performed under local anesthesia, with sedation use when necessary.^10^ They do not impair the visual field and may reduce the risk of high myopia, especially in zone I disease.^11^^,^^12^ Anti-VEGF therapy may also allow vascularization of the peripheral avascular retina, whereas laser permanently ablates the peripheral avascular retina.^12^ Still, anti-VEGF agents have shown a higher risk of ROP recurrence compared with laser photocoagulation.^13^

Moreover, pharmacokinetic studies in preterm infants have shown that serum VEGF levels can be suppressed for up to 2 months after bevacizumab IVT.^14^^,^^15^ However, VEGF is crucial for normal angiogenesis and organ development in preterm infants.^16^ Besides, the systemic administration of anti-VEGF agents for cancer therapy can result in hemorrhages, thromboses, and the onset of heart failure.^17^ There are limited clinical safety data on preterm infants treated with intravitreal anti-VEGF agents, particularly with regard to long-term systemic adverse effects. Some retrospective studies have reported a higher risk of neurodevelopmental disorders in infants treated with anti-VEGF agents compared with laser treatment.^18^, ^19^, ^20^ However, the results remain controversial, primarily due to relatively small sample sizes, short follow-up periods for infants (18‑26 months), and potential indication bias.^18^, ^19^, ^20^, ^21^, ^22^, ^23^ Other adverse effects, such as pulmonary arterial hypertension, have been suspected based on animal studies, but have not yet been confirmed in clinical settings.^24^^,^^25^ To date, very few large-scale observational studies have been conducted to investigate the systemic safety of intravitreal anti-VEGF agents in preterm infants.^11^^,^^25^ This nationwide pharmacoepidemiological study aimed to assess the association between the use of intravitreal anti-VEGF agents in very preterm neonates and the occurrence of neurodevelopmental, CNS, and cardiovascular disorders and the need for vitreoretinal surgery.

## Methods

This study adhered to the tenets of the Declaration of Helsinki, the European General Data Protection Regulation, and the STrengthening the Reporting of OBservational studies in Epidemiology (STROBE) reporting guidelines. As we used anonymized data from a medico-administrative database, patient consent was not required. According to the decree on the French National Health Data System (Système National des Données de Santé [SNDS]) dated June 29, 2021,^26^ prior authorization from an ethics committee was not required for French university hospitals or Regional Health Observatories.

### Study Design

This retrospective nationwide cohort study was conducted using data from the SNDS.

### Data Source

In France, the SNDS is a medico-administrative database which records all reimbursements for out-of-hospital health care, including visits, procedures, and drugs. It also collects administrative and medical data on every hospital stay from private and public health care facilities (the French National Hospital Discharge Database or Programme Médicalisé des Systèmes d'Information). Diagnoses are coded according to the 10th Revision of the International Statistical Classification of Diseases and Related Health Problems, and procedures according to the French Common Classification of Medical Procedures. The SNDS also collects data on the medical causes of death from death certificates (the Epidemiology Centre on Medical Causes of Death). The SNDS covers around 99% of the French population (68 million inhabitants) and undergoes regular validity and reliability assessments. It has been widely used in pharmacoepidemiological studies.^27^, ^28^, ^29^, ^30^

### Cohort Selection and Exposure

All newborns classified as very preterm (gestational age <32 weeks) who were born between January 1, 2015 and December 31, 2022 and treated with laser photocoagulation (BGNP005), cryotherapy (BGLP001), or intravitreal anti-VEGF agents within 24 weeks of birth were included. Intravitreal use of anti-VEGF was identified in the Programme Médicalisé des Systèmes d'Information using the French Common Classification of Medical Procedures code BGLB001 for IVT and/or a corresponding anti-VEGF dispensation using French Common Dispensing Unit and Presentation Identifying Codes (Table S1, available at www.ophthalmologyscience.org). Same-sex twins were excluded as the SNDS database does not permit individual identification of twins. The primary exposure was the type of treatment for ROP: intravitreal anti-VEGF agents versus laser and/or cryotherapy. Patients were followed from birth to 6 years of age and were considered lost to follow-up if no health care utilization was recorded in the SNDS for ≥2 years.

### Clinical Characteristics

Maternal and infant characteristics were collected. Maternal sociodemographic characteristics included age at delivery. Maternal medical characteristics included pre-existing diabetes or hypertension before pregnancy; obstetric conditions such as gestational diabetes, pre-eclampsia, eclampsia, premature rupture of the membranes, placenta previa, retroplacental hematoma, and chorioamnionitis; obstetric trauma; folic acid supplementation; indicators of alcohol and tobacco consumption; history of other psychiatric disorders; dispensing of psychotropic medications and sodium valproate; mode of delivery (vaginal or cesarean section); level of care at the maternity hospital; and length of stay in the maternity ward (Table S1).

The sociodemographic characteristics of the infants included sex, month and year of birth, Complementary Health Solidarity (Complémentaire Santé Solidaire; formerly Couverture Maladie Universelle Complémentaire) coverage, department, and municipality of residence. The latter 2 were used to retrieve the social deprivation index (French DEPrivation Index).^31^ Medical characteristics of the infants included gestational age, birthweight, neonatal conditions related to pregnancy and delivery, maternal conditions affecting the infant, complications of pregnancy, labor and delivery, congenital infectious diseases, neonatal tetanus, neonatal convulsions, ventilatory support, cardiovascular disorders originating in the perinatal period, cerebral disorders of the newborn, severe intraventricular hemorrhage, necrotizing enterocolitis, and postnatal age at the time of ROP treatment (Table S1).

### Main Outcomes

The main outcome we examined was the occurrence of early neurodevelopmental disorders within the first 6 years of life, which were identified through: (1) International Statistical Classification of of Diseases and Related Health Problems codes associated with hospitalizations or long-term illness status (Affection Longue Durée), such as pervasive developmental disorders, including autism spectrum disorders, intellectual disability, disorders of psychological developmental, including cognitive functions disorders, and behavioral and emotional disorders; (2) utilization of outpatient care, including speech therapy sessions and consultations with orthoptists or psychiatrists; and (3) dispensing of neuropsychiatric treatments (antipsychotics, anxiolytics, antidepressants, and psychostimulants) (Table S1). These indicators were defined and validated in a study conducted by EPI-PHARE, which used the SNDS to assess the risk of early neurodevelopmental disorders associated with in utero exposure to valproate.^32^

Secondary outcomes were:

- The occurrence of cardiovascular diseases within the first 6 years of life;
- The occurrence of CNS disorders within the first 6 years of life;
- All-cause mortality within the first year of life, identified using the Epidemiology Centre on Medical Causes of Death data;
- All-cause rehospitalization within the first year of life, identified by any admission in an acute care facility using Programme Médicalisé des Systèmes d'Information data; and
- Surgery for retinal detachment or vitreous hemorrhage within the first year of life (Table S1).

### Statistical Analysis

Continuous variables with a normal distribution are expressed as means (standard deviation), whereas categorical variables are expressed as numbers and percentages.

The primary analysis was conducted on a cohort restricted to newborns born between 2015 and 2019, to ensure a follow-up duration of 6 years. The primary analysis only considered newborns for whom mother‑infant linkage was available in the SNDS to consider maternal characteristics that could have influenced the course of pregnancy and the conditions of birth. To assess the robustness of the findings, sensitivity analyses were performed using alternative definitions of the study cohort.

- Newborns born between 2015 and 2019, with or without available mother‑infant linkage in the SNDS, were included to increase the sample size while ensuring a 6-year follow-up period (sensitivity analysis 1).
- Newborns born between 2015 and 2022, with or without available mother‑infant linkage in the SNDS, were included to increase the sample size further. A 3-year follow-up was conducted, restricting the assessment of outcomes to the first 3 years of life (sensitivity analysis 2).
- Newborns born between 2015 and 2022 for whom mother‑infant linkage was available in the SNDS were included to consider maternal characteristics. A 3-year follow-up period was used to assess the outcomes (sensitivity analysis 3).

We used an inverse probability treatment weighting (IPTW)‑adjusted logistic regression model to compare the outcomes of infants exposed to anti-VEGF agents with those exposed to laser photocoagulation and/or cryotherapy. The variables included in the propensity score were identified through logistic regression and comprised postnatal age at the time of ROP treatment and all pre-exposure sociodemographic and medical characteristics of the mother or infant that significantly impacted the likelihood of the infant being treated with intravitreal anti-VEGF agents. The balance of the covariates between the exposure groups was assessed before and after IPTW using absolute standardized mean differences. Differences of <0.10 indicated good balance of the covariates. To evaluate the robustness of our results, we conducted a sensitivity analysis in the primary analysis population, modifying the set of covariates included in the propensity score model, including newborn age and birthweight in addition to covariates identified through logistic regression as significantly associated with the likelihood of the infant receiving intravitreal anti-VEGF agents (sensitivity analysis 4). The tests were 2-tailed, and statistical significance was set at *P* < 0.05. The analyses were carried out using Statistical Analysis System (SAS) software (version 9.4; SAS Institute).

## Results

Between January 1, 2015 and December 31, 2022, a total of 465 infants were treated for ROP, with anti-VEGF agents (n = 225), laser (n = 187), cryotherapy (n = 5), laser and cryotherapy (n = 1), or with anti-VEGF agents and laser and/or cryotherapy (n = 47). The number of infants treated exclusively with anti-VEGF agents increased by 300% during this period. Anti-VEGF therapy became the predominant treatment in 2018 (Table 1).

**Table 1:** Number of Very Preterm Newborns Treated for Retinopathy of Prematurity from January 2015 to December 2022

Type of Treatment	2015	2016	2017	2018	2019	2020	2021	2022	Total
Anti-VEGF agents	10	18	18	30	24	39	46	40	225
Laser photocoagulation	13	22	19	18	23	33	21	38	187
Cryotherapy	0	1	0	2	0	1	1	0	5
Laser photocoagulation and cryotherapy	0	0	0	0	0	0	0	1	1
Anti-VEGF agents and laser photocoagulation and/or cryotherapy	1	4	2	7	5	10	12	6	47
Total	24	45	39	57	52	83	80	85	465

The primary analysis included 102 infants in total (Fig 1 provides the flowchart). Fifty-nine were treated with anti-VEGF agents and 43 with laser and/or cryotherapy. Of these, 45 (44.1%) were females. The mean gestational age was 25.5 ± 2.0 weeks, the mean birthweight was 733 ± 24 g, and the mean length of stay in hospital was 71.7 ± 46.0 days. The clinical characteristics of these infants before and after IPTW are presented in Table 2. Of the 59 infants treated with anti-VEGF agents, data on the specific agent used were available for 21 infants, all of whom were treated with bevacizumab.

**Figure 1:**
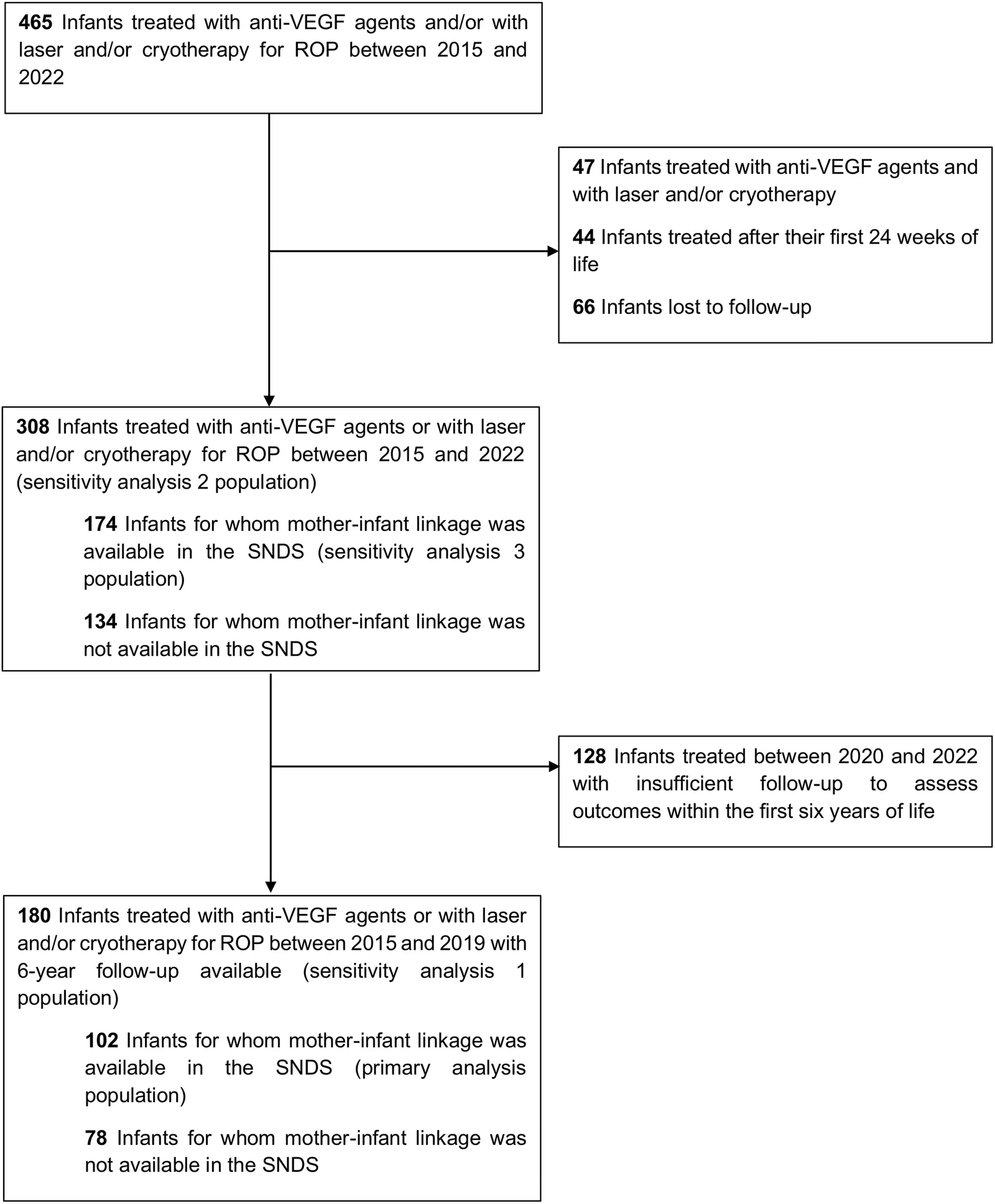
Cohort selection flowchart: primary and sensitivity analysis populations. ROP = retinopathy of prematurity; SNDS = Système National des Données de Santé.

**Table 2:** Baseline Characteristics of Very Preterm Newborns Treated for Retinopathy of Prematurity from January 2015 to December 2019 for Whom Mother‑Infant Linkage was Available in the French National Health Data System before and after Weighting (Primary Analysis Population)

	Before Weighting			After Weighting∗		
	Anti-VEGF Agents (n = 59)	Laser Photocoagulation and/or Cryotherapy (n = 43)	ASD	Anti-VEGF Agents	Laser Photocoagulation and/or Cryotherapy	ASD
Newborn characteristics						
Gestational age at birth (weeks of amenorrhea)	25.6 ± 2.2	25.4 ± 1.7	0.09	25.7 ± 2.4	25.3 ± 1.6	0.16
Female	27 (45.8%)	18 (41.9%)	0.04	24 (43.9%)	15 (42.5%)	0.01
Birthweight, g	741 ± 294	720 ± 160	0.07	758 ± 314	703 ± 166	0.19
Postnatal age at the time of ROP treatment, days	47.0 ± 45.0	58.5 ± 50.8	0.25	51.8 ± 47.0	54.2 ± 49.7	0.05
Intrauterine hypoxia	13 (22.0%)	15 (34.9%)	0.13	13 (24.4%)	11 (30.3%)	0.06
Birth asphyxia	22 (37.3%)	17 (39.5%)	0.02	20 (36.3%)	12 (35.2%)	0.01
Respiratory distress of newborn	54 (91.5%)	40 (93.0%)	0.02	50 (90.3%)	32 (92.2%)	0.02
Ventilatory support	56 (94.9%)	42 (97.7%)	0.03	52 (93.9%)	34 (98.3%)	0.04
Infectious congenital diseases	11 (18,6%)	4 (9.3%)	0.09	10 (18.7%)	3 (7.6%)	0.11
Cardiovascular disorders originating in the perinatal period	27 (45.8%)	17 (39.5%)	0.06	24 (44.5%)	15 (43.2%)	0.01
Disturbances of cerebral status of newborn	4 (6.8%)	1 (2.3%)	0.04	3 (4.9%)	1 (3.4%)	0.01
Severe intraventricular hemorrhage	18 (30.5%)	6 (14.0%)	0.17	13 (23.5%)	8 (22.7%)	0.01
Necrotizing enterocolitis	8 (13.6%)	4 (9.3%)	0.04	7 (12.4%)	3 (7.3%)	0.05
Beneficiary of the French Complementary Health Solidarity	19 (32.2%)	15 (34.9%)	0.03	18 (33.5%)	12 (35.1%)	0.02
High social deprivation index (fifth quintile)†	20 (40.8%)	11 (26.8%)	0.14	23 (42.4%)	10 (27.3%)	0.15
Delivery characteristics						
Cesarean section	38 (64.4%)	20 (47.6%)	0.17	33 (60.6%)	18 (51.2%)	0.09
Level 3 maternity hospital	50 (84.8%)	36 (83.7%)	0.01	45 (81.5%)	30 (85.6%)	0.04
Length of stay in maternity hospital, days	72.0 ± 50.6	71.1 ± 41.2	0.02	67.9 ± 50.2	74.0 ± 39.1	0.12
Maternal characteristics						
Maternal age at delivery, yrs	30.6 ± 6.5	30.2 ± 5.3	0.06	30.6 ± 6.4	30.4 ± 5.1	0.03
Pre-existing hypertension before pregnancy	4 (6.8%)	1 (2.3%)	0.04	4 (6.5%)	2 (7.0%)	0.01
Pre-eclampsia	13 (22.0%)	2 (4.7%)	0.17	8 (14.6%)	4 (12.5%)	0.02
Gestational diabetes	8 (13.6%)	2 (4.7%)	0.09	8 (13.7%)	1 (3.8%)	0.10
Premature rupture of membranes	2 (3.4%)	3 (7.0%)	0.04	1 (2.6%)	2 (5.3%)	0.03
Placenta previa	1 (1.7%)	3 (7.0%)	0.05	1 (2.5%)	2 (5.7%)	0.03
Retroplacental hematoma	7 (11.9%)	5 (11.6%)	0.00	7 (11.9%)	3 (9.3%)	0.03
Chorioamnionitis	11 (18.6%)	7 (16.3%)	0.02	12 (21.5%)	5 (15.5%)	0.06
Folic acid supplementation	21 (35.6%)	17 (39.5%)	0.04	20 (35.7%)	14 (40.3%)	0.05
Dispensing of psychotropic medications in the year prior to pregnancy	13 (22.0%)	8 (18.6%)	0.03	13 (23.2%)	6 (18.6%)	0.05

ASD = absolute standardized difference; ROP = retinopathy of prematurity.

∗ Weighting on a propensity score accounting for postnatal age at the time of ROP treatment, pre-eclampsia, and severe intraventricular hemorrhage.

† Missing data for n = 12 newborns.

Newborns treated with anti-VEGF agents were compared with those treated with laser and/or cryotherapy, using IPTW based on a propensity score that accounted for postnatal age at the time of ROP treatment, pre-eclampsia, and severe intraventricular hemorrhage. Early neurodevelopmental disorders were not observed more frequently in infants treated with anti-VEGF agents than in those treated with laser and/or cryotherapy (71.4% vs. 58.1%; odds ratio [OR], 1.4; 95% confidence interval [CI], 0.8–2.4; *P* = 0.31) (Table 3). No difference was found in terms of CNS disorders at 6 years and all-cause mortality or surgery for retinal detachment or vitreous hemorrhage at 1 year. Cardiovascular diseases at 6 years were observed less frequently in the anti-VEGF group than in the laser/cryotherapy group (7.1% vs. 11.6%; OR, 0.4; 95% CI, 0.1–0.9; *P* = 0.03). All-cause rehospitalization at 1 year was observed less frequently in the anti-VEGF group than in the laser/cryotherapy group (58.9% vs. 72.1%; OR, 0.5; 95% CI, 0.3–0.9; *P* = 0.02) (Table 3).

**Table 3:** Comparison of Systemic and Ophthalmic Outcomes between Infants Treated with Anti-VEGF Agents versus Infants Treated with Laser Photocoagulation and/or Cryotherapy for Retinopathy of Prematurity from 2015 to 2019 and for Whom Mother‑Infant Linkage was Available in the French National Health Data System (n = 102)

Outcome	Time Period	Anti-VEGF Agents (n = 59)	Laser Photocoagulation and/or Cryotherapy (n = 43)	OR (95% CI)∗	*P* Value
Early neurodevelopmental disorders†	Within the first 6 years of life	40 (71.4%)	25 (58.1%)	1.4 (0.8–2.4)	0.31
Central neurological disorders†	Within the first 6 years of life	6 (10.7%)	3 (7.0%)	1.4 (0.5–3.6)	0.55
Cardiovascular diseases†	Within the first 6 years of life	4 (7.1%)	5 (11.6%)	0.4 (0.1–0.9)	0.03
All-cause mortality	Within the first year of life	3 (5.1%)	0 (0.0%)	NA	0.92
All-cause rehospitalization	Within the first year of life	33 (58.9%)	31 (72.1%)	0.5 (0.3–0.9)	0.02
Surgery for retinal detachment or vitreous hemorrhage†	Within the first year of life	6 (10.7%)	3 (7.0%)	1.3 (0.5–3.3)	0.59

CI = confidence interval; NA = not applicable; OR = odds ratio.

∗ Weighting on a propensity score accounting for postnatal age at the time of ROP treatment, pre-eclampsia, and severe intraventricular hemorrhage.

† Analyses were performed on 56 infants treated with anti-VEGF and 43 infants treated with laser and cryotherapy after exclusion of infants lost to deceased infants.

Sensitivity analyses using alternative definitions of the study cohort were performed (Tables S2–S7, available at www.ophthalmologyscience.org). In sensitivity analysis 1, CNS disorders at 6 years were observed more frequently in the anti-VEGF group than in the laser/cryotherapy group (17.0% vs. 6.2%; OR, 2.5; 95% CI, 1.2 – 5.0) (Table S3). No other differences were observed for any of the outcomes in the other sensitivity analyses (Tables S5 and S7).

Results were consistent in the sensitivity analysis 4 which modified the covariates included in the propensity score model (Tables S8 and S9, available at www.ophthalmologyscience.org).

## Discussion

In this nationwide retrospective cohort study, we identified a total of 465 infants treated for ROP between 2015 and 2022. The number of infants treated exclusively with anti-VEGF agents increased by 300% during this period, indicating a widespread adoption of anti-VEGF use in newborns diagnosed with ROP (Table 1). Meanwhile, the number of singleton preterm live births in France decreased from about 44 000 in 2015 to 38 000 in 2022,^33^ of which about 20% were very preterm.^34^^,^^35^

Early neurodevelopmental disorders within the first 6 years of life were frequently observed in our population of very preterm infants, aligning with previous findings,^36^ but the risk did not differ in infants treated with anti-VEGF and in those treated with laser and/or cryotherapy. Results were consistent across sensitivity analyses. This is consistent with a recent meta-analysis reporting no difference in neurodevelopmental outcomes between infants with ROP treated with anti-VEGF agents and those treated with laser therapy.^23^ However, this meta-analysis suggested a higher mortality risk in the anti-VEGF group, largely driven by observational studies. This signal may reflect residual confounding, particularly indication bias. In our study, the number of deaths at 1 year was very small, and larger studies with long-term follow-up are needed to better assess this outcome.

Our analyses showed no increased risk of cardiovascular diseases during the first 6 years of life associated with anti-VEGF agents. This finding is consistent with that of Nitkin et al,^25^ who performed a retrospective cohort study of 982 newborns treated for ROP with anti-VEGF or laser. They did not find a significant association between anti-VEGF therapy and pulmonary hypertension during the hospitalization which included ROP treatment, which occurred at approximately 3 to 4 months of age. In our primary analysis, cardiovascular diseases at 6 years were observed more frequently in the laser group than in the anti-VEGF group, but this was not replicated in sensitivity analyses, likely reflecting random variation.

In one sensitivity analysis, CNS disorders at 6 years were observed more frequently in the anti-VEGF group than in the laser/cryotherapy group. However, this finding was not replicated in the primary or other sensitivity analyses, likely due to random variation.

In our primary analysis, we observed a lower rate of all-cause rehospitalization at 1 year in the anti-VEGF group than in the laser/cryotherapy group, which may be partly driven by procedure-related hospitalizations required for laser or cryotherapy treatment.

In the primary analysis, surgery for retinal detachment or vitreous hemorrhage within the first year of life was similar in infants treated with anti-VEGF and those treated with laser and/or cryotherapy. This contrasts with previous studies reporting a reduced need for surgical interventions with anti-VEGF compared with laser therapy.^11^ Anti-VEGF may have only a transient effect on ROP, especially with short half-life agents such as ranibizumab, allowing ROP to reactivate several weeks or months later. Regardless of the treatment administered, ophthalmic monitoring should be continued until 75 weeks’ postmenstrual age.^12^^,^^37^

Our study has several limitations. First, despite the study’s nationwide design, the number of infants included for the primary analysis remained relatively small (n = 102), which restricted our statistical power. The primary analysis included only newborns for whom the mother‑infant linkage was available in the database, to account for all potential maternal confounders, which excluded >40% of the newborns. Additionally, the primary study population was restricted to infants for whom a 6-year follow-up was available. This excluded infants who were more recently treated with anti-VEGF, a treatment that has become more common in recent years. Yet, this is one of the largest observational studies to date on neurodevelopmental disorders in very premature newborns treated with anti-VEGF compared with laser and/or cryotherapy.^37^^,^^38^ Second, due to the small sample size, significant residual covariate imbalances were still observed between the exposure groups despite applying IPTW. However, several sensitivity analyses were performed using different cohort definitions and different propensity scores, and results were similar overall. Ideally, the severity and zone of ROP would have been included in the propensity score model to ensure adequate balance between treatment groups. However, data on ROP severity and zone were not available in the SNDS database. To mitigate this limitation, we added postnatal age at the time of ROP treatment and several proxy variables and established risk factors for ROP, such as ventilatory support, necrotizing enterocolitis, and severe intraventricular hemorrhage, within the propensity score model. Third, data on the specific anti-VEGF agent administered were available for less than half of the infants in our cohort. All of those infants received bevacizumab, which prevented us from comparing the safety profiles of different types of anti-VEGF agents. Ranibizumab and aflibercept may have a more favorable safety profile than bevacizumab in treating newborns with ROP due to their shorter systemic half-life.^39^ Additionally, our study could not retrieve the anti-VEGF dosing regimens. Lower intravitreal doses of bevacizumab have been shown to be effective and perhaps safer in recent years.^40^ Fourth, our study did not allow us to determine the exact number of anti-VEGF IVTs administered to each infant or assess the need for retreatment.

Our study has several strengths. First, it was the first nationwide study to investigate anti-VEGF agents in newborns with ROP using our medico-administrative database covering the entire French population of about 68 million inhabitants. Second, our study is among the first to provide an extended 6-year follow-up of infants treated with anti-VEGF agents. This allowed us to study neurodevelopmental outcomes often diagnosed after the age of 3,^41^^,^^42^ in contrast to most published studies, which generally limit follow-up to approximately 2 years.^11^ We used validated indicators to comprehensively assess the risk of early neurodevelopmental disorders.^32^ Third, the SNDS database enabled us to analyze neurological, cardiovascular, ophthalmic, and mortality outcomes. Fourth, our methodology was robust, using IPTW and multiple sensitivity analyses with varying cohort definitions and covariates in the propensity score model. To our knowledge, this is to date one of the largest observational studies to evaluate systemic complications, including neurodevelopmental disorders, associated with intravitreal anti-VEGF agents in very premature newborns, with the longest reported follow-up.^37^^,^^38^

## Conclusions

The use of intravitreal anti-VEGF agents to treat ROP in very preterm newborns does not seem to increase the risk of early neurodevelopmental disorders within the first 6 years of life. Although a small excess risk cannot be excluded, these findings might reassure clinicians, especially given the significant increase in the use of anti-VEGF agents to treat ROP in recent years. Further studies with larger sample sizes are warranted.

## References

[1] Shah P.K., Prabhu V., Karandikar S.S. (2016). Retinopathy of prematurity: past, present and future. World J Clin Pediatr.

[2] Nair A., El Ballushi R., Anklesaria B.Z. (2022). A review on the incidence and related risk factors of retinopathy of prematurity across various countries. Cureus.

[3] Ludwig C.A., Chen T.A., Hernandez-Boussard T. (2017). The epidemiology of retinopathy of prematurity in the United States. Ophthalmic Surg Lasers Imaging Retina.

[4] Holmström G., Tornqvist K., Al-Hawasi A. (2018). Increased frequency of retinopathy of prematurity over the last decade and significant regional differences. Acta Ophthalmol.

[5] Andersen C.C., Phelps D.L. (2000). Peripheral retinal ablation for threshold retinopathy of prematurity in preterm infants. Cochrane Database Syst Rev.

[6] Dobson V., Quinn G.E., Summers C.G. (2011). Grating visual acuity results in the early treatment for retinopathy of prematurity study. Arch Ophthalmol.

[7] Mintz-Hittner H.A., Kennedy K.A., Chuang A.Z. (2011). Efficacy of intravitreal bevacizumab for stage 3+ retinopathy of prematurity. N Engl J Med.

[8] Stahl A., Lepore D., Fielder A. (2019). Ranibizumab versus laser therapy for the treatment of very low birthweight infants with retinopathy of prematurity (RAINBOW): an open-label randomised controlled trial. Lancet.

[9] Stahl A., Sukgen E.A., Wu W.C. (2022). Effect of intravitreal aflibercept vs laser photocoagulation on treatment success of retinopathy of prematurity: the FIREFLEYE randomized clinical trial. JAMA.

[10] VanderVeen D.K., Cataltepe S.U. (2019). Anti-vascular endothelial growth factor intravitreal therapy for retinopathy of prematurity. Semin Perinatol.

[11] Wang W.D., Yao Y.H., Lan C.H. (2026). Impact of anti-VEGF therapy versus laser therapy on mortality and treatment outcomes in retinopathy of prematurity: a systematic review and meta-analysis. Acta Ophthalmol.

[12] Hartnett M.E., Stahl A. (2023). Laser versus Anti-VEGF: a paradigm shift for treatment-warranted retinopathy of prematurity. Ophthalmol Ther.

[13] Dablouk M., Chhabra A., Masoud A.T. (2024). Recurrence of retinopathy of prematurity following anti-vascular endothelial growth factor (Anti-VEGF) therapy: a systematic review and meta-analysis. Cureus.

[14] Wu W.C., Lien R., Liao P.J. (2015). Serum levels of vascular endothelial growth factor and related factors after intravitreous bevacizumab injection for retinopathy of prematurity. JAMA Ophthalmol.

[15] Wu W.C., Shih C.P., Lien R. (2017). Serum vascular endothelial growth factor after Bevacizumab or ranibizumab treatment for retinopathy of prematurity. Retina.

[16] VanderVeen D.K., Melia M., Yang M.B. (2017). Anti-Vascular endothelial growth factor therapy for primary treatment of type 1 retinopathy of prematurity: a report by the American Academy of ophthalmology. Ophthalmology.

[17] Kamba T., McDonald D.M. (2007). Mechanisms of adverse effects of anti-VEGF therapy for cancer. Br J Cancer.

[18] Morin J., Luu T.M., Superstein R. (2016). Neurodevelopmental outcomes following Bevacizumab injections for retinopathy of prematurity. Pediatrics.

[19] Natarajan G., Shankaran S., Nolen T.L. (2019). Neurodevelopmental outcomes of preterm infants with retinopathy of prematurity by treatment. Pediatrics.

[20] Arima M., Akiyama M., Fujiwara K. (2020). Neurodevelopmental outcomes following intravitreal bevacizumab injection in Japanese preterm infants with type 1 retinopathy of prematurity. PLoS One.

[21] Lien R., Yu M.H., Hsu K.H. (2016). Neurodevelopmental outcomes in infants with retinopathy of prematurity and Bevacizumab treatment. PLoS One.

[22] Fan Y.Y., Huang Y.S., Huang C.Y. (2019). Neurodevelopmental outcomes after intravitreal bevacizumab therapy for retinopathy of prematurity: a prospective case-control Study. Ophthalmology.

[23] Wang W.D., Yao Y.H., Lan C.H. (2026). Impact of anti-VEGF therapy versus laser therapy on mortality and treatment outcomes in retinopathy of prematurity: a systematic review and meta-analysis. Acta Ophthalmol.

[24] Demir C., Karaman M., Uçan E.S. (2021). Effects of bevacizumab administration on the hypoxia - induced pulmonary hypertension rat model. Turk J Med Sci.

[25] Nitkin C.R., Bamat N.A., Lagatta J. (2022). Pulmonary hypertension in preterm infants treated with laser vs anti-vascular endothelial growth factor therapy for retinopathy of prematurity. JAMA Ophthalmol.

[26] (2021). Décret n° 2021-848 du 29 juin 2021 relatif au traitement de données à caractère personnel dénommé « système national des données de santé.

[27] Maillard O., Bun R., Laanani M. (2024). Use of the French National Health Data System (SNDS) in pharmacoepidemiology: a systematic review in its maturation phase. Therapie.

[28] Luu M., Benzenine E., Barkun A. (2019). Safety of first year vaccination in children born to mothers with inflammatory bowel disease and exposed in utero to anti-TNFα agents: a French nationwide population-based cohort. Aliment Pharmacol Ther.

[29] Luu M., Benzenine E., Doret M. (2018). Continuous Anti-TNFα use throughout pregnancy: possible complications for the mother but not for the fetus. A retrospective cohort on the French national health insurance database (EVASION). Am J Gastroenterol.

[30] Baudin F., Benzenine E., Mariet A.S. (2018). Association of acute endophthalmitis with intravitreal injections of corticosteroids or anti-vascular growth factor agents in a nationwide Study in France. JAMA Ophthalmol.

[31] Rey G., Jougla E., Fouillet A., Hémon D. (2009). Ecological association between a deprivation index and mortality in France over the period 1997 - 2001: variations with spatial scale, degree of urbanicity, age, gender and cause of death. BMC Public Health.

[32] Coste J., Blotiere P.O., Miranda S. (2020). Risk of early neurodevelopmental disorders associated with in utero exposure to valproate and other antiepileptic drugs: a nationwide cohort study in France. Sci Rep.

[33] DREES (2023).

[34] Torchin H., Ancel P.Y. (2016). Épidémiologie et facteurs de risque de la prématurité. J de Gynécologie Obstétrique Biologie de la Reprod.

[35] Inserm (2023). Prématurité. Des bébés qui arrivent trop tôt.

[36] Song I.G. (2023). Neurodevelopmental outcomes of preterm infants. Clin Exp Pediatr.

[37] Tsai A.S., Chou H.D., Ling X.C. (2022). Assessment and management of retinopathy of prematurity in the era of anti-vascular endothelial growth factor (VEGF). Prog Retin Eye Res.

[38] Kaushal M., Razak A., Patel W. (2021). Neurodevelopmental outcomes following bevacizumab treatment for retinopathy of prematurity: a systematic review and meta-analysis. J Perinatol.

[39] Avery R.L., Castellarin A.A., Steinle N.C. (2017). Systemic pharmacokinetics and pharmacodynamics of intravitreal aflibercept, bevacizumab, and ranibizumab. Retina.

[40] Wallace D.K., Kraker R.T., Freedman S.F. (2020). Short-term outcomes after very low-dose intravitreous bevacizumab for retinopathy of prematurity. JAMA Ophthalmol.

[41] Lipkin P.H., Macias M.M. (2020). Promoting optimal development: identifying infants and young children with developmental disorders through developmental surveillance and screening. Pediatrics.

[42] Johnson S., Marlow N. (2011). Preterm birth and childhood psychiatric disorders. Pediatr Res.

