# Supplementary material for: Neurodevelopmental Disorders in Infants Treated with Anti-VEGF Agents versus Laser Photocoagulation or Cryotherapy for Retinopathy of Prematurity
Source: Ophthalmol Sci. 2026 Aug 17;6(11):101371. doi: 10.1016/j.xops.2026.101371 (PMC13654026; doi:10.1016/j.xops.2026.101371)
Supplement: Supplementary file 1 [file mmc1.pdf]

Table S1. Codes used in the study for variable identification in the French National Health Data System (SNDS) database

Exposure/Outcomes/Characteristics	Indicators	ICD-10 codes	CCAM codes	ATC codes	Other codes
Exposure to anti-VEGF	• Injection of a pharmacological agent in the vitreous		BGLB001		
	• and/or Dispensing of :<br>- Bevacizumab				UCD13 3400890012478, 3400898765444, 3400890012492, 3400890011204, 3400890011211, 3400892611044, 3400892611105, 3400890005234, 3400890005241, 3400890000994, 3400890001007, 3400890012706, 3400890012713, 3400890026512, 3400890026529, 3400890000246, 3400890000253
	- or Aflibercept				UCD 13 3400893949924, 3400893950005, 3400893971116, 3400893953945; CIP 3400926783501, 3400926783679, 3400958418563, 3400958418792

	- or Ranibizumab		UCD 13 3400894009948, 3400890028431 ; CIP 3400927671197, 3400930258675
Early neurodevelopmental disorders	• A long-term condition and/or hospitalization associated with one of the following diagnoses:		
	- Pervasive developmental disorders, including autism spectrum disorders	F84	
	- Intellectual disability	F70-F79	
	- Disorders of psychological development, including cognitive functions disorders	F80-F89	
	- Behavioral and emotional disorders with onset usually occurring in childhood and adolescence	F90-F98	
	• and/or Use of outpatient or ambulatory care, identified by reimbursement of at least:		
	- One speech therapy session		PS5 3132 (AMO)
	- One consultation with an orthoptist		PS5 3133 (AMY)
	- One consultation with a psychiatrist		APY, AVY, CNP (+/-F), PS5 1118, CS

				(+/-F/N) associated with PFS_SPE_COD 33 (General Psychiatry) or 75 (Child and Adolescent Psychiatry)
	• and/or at least one dispensing of neuropsychiatric treatments			
	- Antipsychotics		N05A	
	- Anxiolytics		N05B	
	- Antidepressants		N06A	
	- Psychostimulants		N06B	
Cardiovascular diseases	• A long-term condition and/or hospitalization associated with one of the following diagnoses:			
	- Hypertensive diseases	I10–I15		
	- Ischemic heart diseases	I20–I25		
	- Cardiopulmonary diseases and pulmonary circulation disorders	I26–I28		
	- Left bundle branch block and atrioventricular block	I44		
	- Other conduction disorders	I45		
	- Cardiac arrest	I46		
	- Paroxysmal tachycardia	I47		
	- Atrial fibrillation and flutter	I48		

	- Other cardiac arrhythmias	I49	
	- Heart failure	I50	
	- Complications of heart diseases	I51	
	- Aortic aneurysm and dissection	I71	
	- Other aneuvrysms and dissections	I72	
	• and/or at least one dispensing of cardiovascular treatments		C01
	- Cardiac therapy		C02
	- Antihypertensives		C03
	- Beta blocking agents		C07
	- Calcium channel blockers		C08
	- Agents acting on the renin–angiotensin system		C09
	- Antithrombotic agents		B01
Central neurological disorders	• A long-term condition and/or hospitalization associated with one of the following diagnoses:		
	- Epilepsy	G40	
	- Status epilepticus	G41	
	- Hemiplegia	G81	
	- Paraplegia and tetraplegia	G82	
	- Other paralytic syndromes	G83	
	- Cerebrovascular diseases	I60-I69	

	- Transient cerebral ischaemic attacks and related syndromes	G45		
	- Vascular syndromes of brain in cerebrovascular diseases	G46		
	- Hydrocephalus	G91		
	- Other disorders of brain	G93		
	- Other diseases of spinal cord	G95		
	- Other disorders of central nervous system	G96		
	• and/or at least one dispensing of neurological treatments			
	- Antiepileptics			N03
	- Muscle relaxants			M03
	- Benzodiazepine derivatives, benzodiazepine related drugs			N03AE, N05BA, N05CD, N05CF
Surgery for retinal detachment or vitreous hemorrhage	• Retinal detachments and breaks	H33		
	• and/or one of the following surgical procedures for retinal detachment or vitreous hemorrhage			
	- Vitrectomy via posterior sclerotomy, without retinal coagulation		BGFA001	
	- Vitrectomy with membrane		BGFA003	

	dissection, coagulation, and intraocular tamponade or transscleral indentation, for retinopathy of prematurity	
	- Vitrectomy via posterior sclerotomy, with transscleral retinal coagulation, without tamponade	BGFA006
	- Vitrectomy via posterior sclerotomy, with retinal endocoagulation using laser or diathermy, without tamponade	BGFA009
	- Vitrectomy via posterior sclerotomy, with retinal coagulation and temporary internal tamponade	BGFA010
	- Retinopexy by cryocoagulation or external laser, with scleral indentation over more than one quadrant, with internal gas tamponade	BGDA001
	- Retinopexy by cryocoagulation or external laser, with	BGDA002

	scleral indentation over more than one quadrant, without tamponade	
	- Retinopexy by cryocoagulation or external laser, with scleral indentation limited to one quadrant, with internal gas tamponade	BGDA003
	- Retinopexy by cryocoagulation or external laser, with scleral indentation limited to one quadrant, without tamponade	BGDA004
	- Retinopexy by cryocoagulation or external laser, with 360° scleral indentation (encircling band), without tamponade	BGDA005
	- Retinopexy by cryocoagulation or external laser, with 360° scleral indentation (encircling band), with internal gas tamponade	BGDA006
	- Transscleral balloon indentation	BGDA007

	- Retinopexy by cryocoagulation or external laser, without indentation, with internal gas tamponade	BGDA008
	- Vitreoretinal surgery combining indentation and at least 4 of the following procedures: coagulation, vitrectomy, laser endocoagulation, retinotomy, internal tamponade, fluid-gas exchange, membrane dissection	BGMA001
	- Vitreoretinal surgery combining at least 4 of the following procedures: coagulation, vitrectomy, laser endocoagulation, retinotomy, internal tamponade, fluid-gas exchange, membrane dissection, retinectomy	BGMA002
	- Modification of scleral indentation	BGMA003

	for persistent retinal detachment		
Maternal medical characteristics	• Preexisting diabetes*	E10-E14, G59.0, G63.2, G73.0, G99.0, H28.0, H36.0, I79.2, L97, M14.2, M14.6, N08.3	ATC2A10, excluding drugs prescribed for indications other than diabetes
	• Preexisting hypertension*		C02AB02, C02AC01, C02AC02, C02AC05, C02AC06, C02CA01, C02CA06, C02DC01, C02LA01, C03AA01, C03AA03, C03BA04, C03BA10, C03BA11, C03BX03, C03CA01, C03CA02, C03CA03, C03DA01, C03DB01, C03EA01,

		C03EA04,<br>C07AA02,<br>C07AA03,<br>C07AA05,<br>C07AA06,<br>C07AA12,<br>C07AA15,<br>C07AA16,<br>C07AA23,<br>C07AB02,<br>C07AB03,<br>C07AB04,<br>C07AB05,<br>C07AB07,<br>C07AB08,<br>C07AB12,<br>C07AG01,<br>C07BA02,<br>C07BB02,<br>C07BB03,<br>C07BB07,<br>C07BB12,<br>C07CA03,<br>C07DA06,<br>C07FB02,<br>C07FB03,<br>C08CA01,<br>C08CA02,<br>C08CA03,<br>C08CA04,<br>C08CA05,<br>C08CA08,<br>C08CA09,<br>C08CA11,<br>C08CA13,<br>C08CX01,<br>C08DA01,

	C08DB01, C08GA02, C09AA01, C09AA02, C09AA03, C09AA04, C09AA05, C09AA06, C09AA07, C09AA08, C09AA09, C09AA10, C09AA13, C09AA15, C09AA16, C09BA01, C09BA02, C09BA03, C09BA04, C09BA05, C09BA06, C09BA07, C09BA09, C09BA15, C09BB02, C09BB04, C09BB07, C09BB10, C09BX02, C09CA01, C09CA02, C09CA03, C09CA04, C09CA06, C09CA07, C09CA08, C09DA01,

			C09DA02,<br>C09DA03,<br>C09DA04,<br>C09DA06,<br>C09DA07,<br>C09DA08,<br>C09DB01,<br>C09DB02,<br>C09DB04,<br>C09XA02,<br>C09XA52,<br>C10BX03
	• Obstetric conditions<br>- Gestational diabetes<br>- Preeclampsia<br><br>- Eclampsia<br>- Premature rupture of membranes<br>- Placenta previa<br>- Retroplacental hematoma<br>- Chorioamnionitis<br>- Obstetric trauma	 O244<br>O11,<br>O14<br>O15<br>O42<br>O44<br>O45<br>O411<br>P10-<br>P15	
	• Folic acid supplementation from one month prior to pregnancy through the end of the first trimester		B03BB01
	• Indicator of alcohol consumption in the year prior to or during pregnancy	F10	
	• Indicator of tobacco use in the year prior to or during pregnancy	F17	
	• History of other psychiatric disorders: long-term	F (except	

	condition and/or hospitalization associated with a mental or behavioural disorder	F10 and F17)		
	• Dispensing of psychotropic medications in the year prior to or during pregnancy*		N03AG01,<br>N03AG02,<br>N05A (excluding N05AL06),<br>N05BA01,<br>N05BA04,<br>N05BA05,<br>N05BA06,<br>N05BA08,<br>N05BA09,<br>N05BA11,<br>N05BA12,<br>N05BA16,<br>N05BA18,<br>N05BA21,<br>N05BA23,<br>N05BB01,<br>N05BB02,<br>N05BC01,<br>N05CB02,<br>N05BC51,<br>N05BE01,<br>N05BX03,<br>N05CD03,<br>N05CD04,<br>N05CD05,<br>N05CD06,<br>N05CD07,<br>N05CD11,<br>N05CF01,<br>N05CF04,<br>N05CF02,	- For N03AG01: only 3400934876233, 3400934876691, 3400935444271<br>- Excluding 3400932896332, 3400933338022

		N05CM11,<br>N05CM16,<br>N05CX N06A<br>(excluding<br>N06AX12)
	• Dispensing of sodium valproate during pregnancy	N03AG01
	• Mode of delivery<br>• Vaginal delivery<br><br><br><br><br><br><br><br><br><br>• Caesarean section	 JQGD001<br>JQGD002<br>JQGD003<br>JQGD004<br>JQGD005<br>JQGD007<br>JQGD008<br>JQGD010<br>JQGD012<br>JQGD013<br>JQGA002<br>JQGA003<br>JQGA004<br>JQGA005
Neonatal infant medical characteristics	• Level of care of the maternity hospital	Level 1, 2A, 2B, or 3 maternity unit, or facility without a maternity unit, according to the classification of the French Ministry of Health
	• Fetus and newborn affected by maternal factors and by complications of pregnancy, labour and delivery	P00-<br>P04
	• Neonatal conditions related to pregnancy and delivery<br>- Intrauterine hypoxia<br>- Birth asphyxia<br>- Respiratory distress of newborn	 <br>P20<br>P21<br>P22

	• Cardiovascular disorders originating in the perinatal period	P29	
	• Convulsions of newborn	P90	
	• Other disturbances of cerebral status of newborn, including: neonatal cerebral ischemia, acquired periventricular cysts, cerebral leukomalacia, cerebral irritability, decreased cerebral activity, neonatal coma, neonatal anoxic-ischemic encephalopathy, acquired hydrocephalus of the newborn and other cerebral disorders	P91	
	• Congenital infectious diseases	A50, P35, P37	
	• Tetanus neonatorum	A33	
	• Ventilatory support		GLLD004, GLLD005, GLLD006, GLLD008, GLLD009, GLLD012, GLLD013, GLLD015, GLLD016, GLLD017
	• Severe intraventricular hemorrhage	P52.1, P52.2	
	• Necrotizing enterocolitis	P77	
	• Retinopathy of Prematurity	H351	

* Codes defined according to the Medical Methodology for Mapping Diseases and Expenditures, Version G11, of the French National Health Insurance

Abbreviations: ICD-10, International Statistical Classification of Diseases and Related Health Problems, Tenth Revision; CCAM, French Common Classification of Medical Procedures; ATC, Anatomical Therapeutic Chemical Classification System; VEGF, Vascular Endothelial Growth Factor; UCD, Common Dispensing Unit; CIP, Presentation Identifying Codes.
