# Supplementary material for: Neurodevelopmental Disorders in Infants Treated with Anti-VEGF Agents versus Laser Photocoagulation or Cryotherapy for Retinopathy of Prematurity
Source: Ophthalmol Sci. 2026 Aug 17;6(11):101371. doi: 10.1016/j.xops.2026.101371 (PMC13654026; doi:10.1016/j.xops.2026.101371)
Supplement: Supplementary file 2 [file mmc2.pdf]

Table S2. Baseline characteristics of very preterm newborns treated for retinopathy of prematurity from January 2015 to December 2019, with or without mother-infant linkage available in the French National Health Data System before and after weighting (sensitivity analysis 1 population)

	Before weighting			After weighting**		
	**Anti-VEGF agents (n = 98)**	**Laser photocoagulation and/or cryotherapy (n = 82)**	**ASD**	**Anti-VEGF agents**	**Laser photocoagulation and/or cryotherapy**	**ASD**
**Newborn characteristics**						
Gestational age at birth (weeks of amenorrhea)	25.5 ± 1.9	25.5 ± 1.8	0.02	25.6 ± 2.0	25.4 ± 1.7	0.10
Female	41 (41.8%)	39 (47.6%)	0.06	40 (42.2%)	37 (48.8%)	0.07
Birthweight, g	731 ± 266	728 ± 175	0.01	730 ± 272	725 ± 174	0.02
Postnatal age at the time of ROP treatment, days	45.3 ± 43.8	53.8 ± 53.3	0.19	48.6 ± 44.3	48.8 ± 52.1	0.00
Intrauterine hypoxia	19 (19.4%)	27 (32.9%)	0.14	19 (19.9%)	24 (32.0%)	0.12
Birth asphyxia	33 (33.7%)	23 (36.6%)	0.03	31 (33.1%)	26 (34.1%)	0.01
Respiratory distress of newborn	91 (92.9%)	76 (92.7%)	0.00	87 (92.2%)	69 (92.3%)	0.00
Ventilatory support	93 (94.9%)	78 (95.1%)	0.00	89 (94.4%)	71 (95.7%)	0.01
Infectious congenital diseases	13 (13.3%)	10 (12.2%)	0.01	12 (13.3%)	9 (12.4%)	0.01
Cardiovascular disorders originating in the perinatal period	40 (50.0%)	34 (41.5%)	0.09	47 (49.8%)	31 (41.2%)	0.09
Disturbances of cerebral status of newborn	11 (11.2%)	4 (4.9%)	0.06	9 (9.5%)	4 (5.5%)	0.04
Severe intraventricular hemorrhage	33 (33.7%)	12 (14.6%)	0.19	23 (25.0%)	19 (24.9%)	0.00
Necrotizing enterocolitis	15 (15.3%)	10 (12.2%)	0.03	13 (13.9%)	10 (12.9%)	0.01
Beneficiary of the French Complementary Health Solidarity	28 (28.6%)	23 (28.1%)	0.01	27 (29.0%)	21 (28.1%)	0.01

High social deprivation index (5th quintile) *	31 (36.5%)	20 (26.3%)	0.10	35 (36.9%)	20 (26.3%)	0.06
Length of stay in maternity hospital, days	82.2 ± 51.0	81.7 ± 42.1	0.01	79.5 ± 51.4	84.8 ± 40.6	0.10

* Missing data for n = 19 newborns

** Weighting on a propensity score accounting for postnatal age at the time of ROP treatment and severe intraventricular hemorrhage.

Abbreviations: Anti-VEGF, anti-vascular endothelial growth factor; ASD, absolute standardized difference; ROP, retinopathy of prematurity.
