# Supplementary material for: Neurodevelopmental Disorders in Infants Treated with Anti-VEGF Agents versus Laser Photocoagulation or Cryotherapy for Retinopathy of Prematurity
Source: Ophthalmol Sci. 2026 Aug 17;6(11):101371. doi: 10.1016/j.xops.2026.101371 (PMC13654026; doi:10.1016/j.xops.2026.101371)
Supplement: Supplementary file 3 [file mmc3.pdf]

Table S3. Comparison of systemic and ophthalmological outcomes between infants treated with anti-vascular endothelial growth factor agents vs. infants treated with laser photocoagulation and/or cryotherapy for retinopathy of prematurity from 2015 to 2019 (n = 180, sensitivity analysis 1)

Outcome	Time period	Anti-VEGF agents (n = 98)	Laser photocoagulation and/or cryotherapy (n = 82)	OR (95% CI)*	*p*
Early neurodevelopmental disorders	Within the first 6 years of life**	66 (70.2%)	50 (61.7%)	1.4 (0.9 – 2.2)	0.11
Central neurological disorders	Within the first 6 years of life**	16 (17.0%)	5 (6.2%)	2.5 (1.2 – 5.0)	0.01
Cardiovascular diseases	Within the first 6 years of life**	11 (11.7%)	8 (9.9%)	1.2 (0.6 – 2.3)	0.62
All-cause mortality	Within the first year of life	4 (4.1%)	1 (1.2%)	2.8 (0.6 – 13.8)	0.20
All-cause rehospitalization	Within the first year of life**	64 (68.1%)	53 (65.4%)	1.1 (0.7 – 1.8)	0.54
Surgery for retinal detachment or vitreous hemorrhage	Within the first year of life**	9 (9.6%)	5 (6.2%)	2.0 (0.9 – 4.7)	0.11

* Weighting on a propensity score accounting for postnatal age at the time of ROP treatment and severe intraventricular hemorrhage.

** Analyses were performed on 94 infants treated with anti-VEGF and 81 infants treated with laser and cryotherapy after exclusion of infants who died before outcome assessment.

Abbreviations: Anti-VEGF, anti-Vascular Endothelial Growth Factor; ROP, retinopathy of prematurity.
