# Supplementary material for: Neurodevelopmental Disorders in Infants Treated with Anti-VEGF Agents versus Laser Photocoagulation or Cryotherapy for Retinopathy of Prematurity
Source: Ophthalmol Sci. 2026 Aug 17;6(11):101371. doi: 10.1016/j.xops.2026.101371 (PMC13654026; doi:10.1016/j.xops.2026.101371)
Supplement: Supplementary file 4 [file mmc4.pdf]

Table S4. Baseline characteristics of very preterm newborns treated for retinopathy of prematurity from January 2015 to December 2022, with or without mother-infant linkage available in the French National Health Data System before and after weighting (sensitivity analysis 2 population)

	Before weighting			After weighting**		
	**Anti-VEGF agents (n = 189)**	**Laser photocoagulation and/or cryotherapy (n = 119)**	**ASD**	**Anti-VEGF agents**	**Laser photocoagulation and/or cryotherapy**	**ASD**
**Newborn characteristics**						
Gestational age at birth (weeks of amenorrhea)	25.4 ± 1.9	25.4 ± 1.6	0.03	25.4 ± 1.9	25.4 ± 1.6	0.02
Female	80 (42.3%)	54 (45.4%)	0.03	79 (42.5%)	51 (46.1%)	0.04
Birthweight, g	724 ± 242	725 ± 166	0.00	724 ± 246	726 ± 167	0.01
Postnatal age at the time of ROP treatment, days	41.3 ± 43.2	57.2 ± 52.0	0.37	46.5 ± 44.3	45.9 ± 50.2	0.01
Intrauterine hypoxia	38 (20.1%)	38 (31.9%)	0.12	37 (20.0%)	35 (31.5%)	0.12
Birth asphyxia	66 (34.9%)	45 (37.8%)	0.03	65 (34.9%)	41 (37.2%)	0.02
Respiratory distress of newborn	177 (93.7%)	109 (91.6%)	0.02	173 (93.1%)	101 (91.8%)	0.01
Ventilatory support	180 (95.2%)	115 (96.6%)	0.01	176 (95.2%)	107 (96.9%)	0.02
Infectious congenital diseases	26 (13.8%)	18 (15.1%)	0.01	25 (13.4%)	18 (16.1%)	0.03
Cardiovascular disorders originating in the perinatal period	93 (49.2%)	57 (47.9%)	0.01	90 (48.6%)	53 (48.6%)	0.00
Disturbances of cerebral status of newborn	23 (12.2%)	6 (5.0%)	0.07	20 (10.9%)	6 (5.8%)	0.05
Severe intraventricular hemorrhage	62 (32.8%)	24 (20.2%)	0.13	52 (28.1%)	31 (28.6%)	0.00
Necrotizing enterocolitis	26 (13.8%)	17 (14.3%)	0.01	23 (12.7%)	18 (15.9%)	0.03
Beneficiary of the French Complementary Health Solidarity	49 (25.9%)	29 (24.4%)	0.02	48 (25.9%)	27 (24.4%)	0.01
High social deprivation index (5th quintile) *	51 (30.3%)	30 (25.0%)	0.05	57 (30.7%)	27 (24.8%)	0.06

Length of stay in maternity hospital, days	83.4 ± 52.1	80.6 ± 42.7	0.05	81.0 ± 51.9	84.5 ± 43.2	0.07

* Missing data for n = 34 newborns

** Weighting on a propensity score accounting for postnatal age at the time of ROP treatment and severe intraventricular hemorrhage.

Abbreviations: Anti-VEGF, anti-vascular endothelial growth factor; ASD, absolute standardized difference; ROP, retinopathy of prematurity.
