# Supplementary material for: Neurodevelopmental Disorders in Infants Treated with Anti-VEGF Agents versus Laser Photocoagulation or Cryotherapy for Retinopathy of Prematurity
Source: Ophthalmol Sci. 2026 Aug 17;6(11):101371. doi: 10.1016/j.xops.2026.101371 (PMC13654026; doi:10.1016/j.xops.2026.101371)
Supplement: Supplementary file 5 [file mmc5.pdf]

**Table S5.** Comparison of systemic and ophthalmological outcomes between infants treated with anti-vascular endothelial growth factor agents vs. infants treated with laser photocoagulation and/or cryotherapy for retinopathy of prematurity from January 2015 to December 2022 (n = 308, sensitivity analysis 2)

| Outcome | Time period | Anti-VEGF agents (n = 189) | Laser photocoagulation and/or cryotherapy (n = 119) | OR (95% CI)* | *p* |
|---|---|---|---|---|---|
| Early neurodevelopmental disorders | Within the first 3 years of life** | 96 (52.7%) | 58 (49.2%) | 1.2 (0.7 – 2.0) | 0.58 |
| Central neurological disorders | Within the first 3 years of life** | 20 (11.0%) | 10 (8.5%) | 1.8 (0.6 – 5.9) | 0.31 |
| Cardiovascular diseases | Within the first 3 years of life** | 23 (12.6%) | 12 (10.2%) | 1.1 (0.5 – 2.5) | 0.75 |
| All-cause mortality | Within the first year of life | 7 (3.7%) | 1 (0.8%) | 4.5 (0.6 – 37.4) | 0.16 |
| All-cause rehospitalization | Within the first year of life** | 129 (70.9%) | 81 (68.6%) | 1.1 (0.7 – 1.8) | 0.68 |
| Surgery for retinal detachment or vitreous hemorrhage | Within the first year of life** | 15 (8.2%) | 10 (8.5%) | 1.0 (0.4 – 2.2) | 0.94 |

* Weighting on a propensity score accounting for postnatal age at the time of ROP treatment and severe intraventricular hemorrhage.

** Analyses were performed on 182 infants treated with anti-VEGF and 118 infants treated with laser and cryotherapy after exclusion of infants who died before outcome assessment.

Abbreviations: Anti-VEGF, anti-Vascular Endothelial Growth Factor; ROP, retinopathy of prematurity.
