# Supplementary material for: Neurodevelopmental Disorders in Infants Treated with Anti-VEGF Agents versus Laser Photocoagulation or Cryotherapy for Retinopathy of Prematurity
Source: Ophthalmol Sci. 2026 Aug 17;6(11):101371. doi: 10.1016/j.xops.2026.101371 (PMC13654026; doi:10.1016/j.xops.2026.101371)
Supplement: Supplementary file 6 [file mmc6.pdf]

Table S6. Baseline characteristics of very preterm newborns treated for a retinopathy of prematurity from January 2015 to December 2022, for whom mother-infant linkage was available in the French National Health Data System before and after weighting (sensitivity analysis 3 population)

	Before weighting			After weighting**		
	Anti-VEGF agents (n = 111)	Laser photocoagulation and/or cryotherapy (n = 63)	ASD	Anti-VEGF agents	Laser photocoagulation and/or cryotherapy	ASD
**Newborn characteristics**						
Gestational age at birth (weeks of amenorrhea)	25.4 ± 2.1	25.3 ± 1.5	0.05	25.4 ± 2.1	25.3 ± 1.5	0.07
Female	47 (42.3%)	25 (39.7%)	0.03	42 (41.2%)	25 (40.9%)	0.00
Birthweight, g	739 ± 270	727 ± 154	0.04	738 ± 269	725 ± 154	0.05
Postnatal age at the time of ROP treatment, days	43.7 ± 45.4	57.6 ± 49.4	0.31	50.6 ± 47.3	49.7 ± 48.3	0.02
Intrauterine hypoxia	24 (21.6%)	19 (30.2%)	0.09	26 (24.9%)	17 (29.1%)	0.04
Birth asphyxia	41 (36.9%)	24 (38.1%)	0.01	39 (37.6%)	24 (39.6%)	0.02
Respiratory distress of newborn	102 (91.9%)	57 (90.5%)	0.01	95 (91.9%)	54 (89.7%)	0.02
Ventilatory support	106 (95.5%)	62 (98.4%)	0.03	99 (95.8%)	59 (98.6%)	0.03
Infectious congenital diseases	17 (15,3%)	9 (14.3%)	0.01	15 (14.9%)	9 (14.8%)	0.00
Cardiovascular disorders originating in the perinatal period	51 (46.0%)	30 (47.6%)	0.02	49 (47.8%)	27 (45.4%)	0.02
Disturbances of cerebral status of newborn	9 (8.1%)	2 (3.2%)	0.05	8 (7.5%)	2 (2.8%)	0.05
Severe intraventricular hemorrhage	34 (30.6%)	13 (20.6%)	0.10	32 (30.9%)	13 (21.0%)	0.10
Necrotizing enterocolitis	15 (13.5%)	8 (12.7%)	0.01	13 (12.2%)	7 (11.5%)	0.01
Beneficiary of the French Complementary Health Solidarity	30 (27.0%)	16 (25.4%)	0.02	29 (27.7%)	16 (26.0%)	0.02
High social deprivation index (5th quintile) *	34 (30.6%)	16 (25.0%)	0.06	33 (31.7%)	14 (23.4%)	0.05
**Delivery characteristics**						
Caesarean section	64 (57.3%)	29 (46.8%)	0.11	59 (57.1%)	27 (45.3%)	0.01

Level 3 maternity hospital	93 (83.8%)	53 (84.1%)	0.00	86 (83.7%)	50 (83.7%)	0.00
Length of stay in maternity hospital, days	73.4 ± 51.2	70.9 ± 41.6	0.05	71.7 ± 50.2	72.1 ± 42.5	0.01
**Maternal characteristics**						
Maternal age at delivery, years	30.6 ± 6.4	30.1 ± 5.1	0.07	30.7 ± 6.3	30.1 ± 5.1	0.10
Preexisting hypertension before pregnancy	4 (3.6%)	2 (3.2%)	0.00	4 (3.6%)	2 (3.1%)	0.00
Pre-eclampsia	18 (16.2%)	6 (9.5%)	0.07	16 (15.2%)	6 (10.0%)	0.05
Gestational diabetes	14 (12.6%)	6 (9.5%)	0.03	12 (11.8%)	6 (9.6%)	0.02
Premature rupture of membranes	7 (6.3%)	3 (4.8%)	0.02	6 (6.3%)	3 (4.5%)	0.02
Placenta praevia	2 (1.8%)	5 (7.9%)	0.06	6 (5.4%)	3 (4.3%)	0.01
Retroplacental hematoma	10 (9.0%)	7 (11.1%)	0.02	11 (10.8%)	5 (8.6%)	0.02
Chorioamnionitis	23 (20.7%)	11 (17.5%)	0.03	21 (20.4%)	12 (19.4%)	0.01
Folic acid supplementation	51 (46.0%)	26 (41.2%)	0.05	47 (45.9%)	25 (42.2%)	0.04
Dispensing of psychotropic medications in the year prior to pregnancy	17 (15.3%)	10 (15.9%)	0.01	18 (17.2%)	9 (15.5%)	0.02

* Missing data for n = 16 newborns

** Weighting on a propensity score accounting for pre-eclampsia, postnatal age at the time of ROP treatment, and severe intraventricular hemorrhage.

Abbreviations: Anti-VEGF, anti-vascular endothelial growth factor; ASD, absolute standardized difference; ROP, retinopathy of prematurity.
