# Supplementary material for: Neurodevelopmental Disorders in Infants Treated with Anti-VEGF Agents versus Laser Photocoagulation or Cryotherapy for Retinopathy of Prematurity
Source: Ophthalmol Sci. 2026 Aug 17;6(11):101371. doi: 10.1016/j.xops.2026.101371 (PMC13654026; doi:10.1016/j.xops.2026.101371)
Supplement: Supplementary file 7 [file mmc7.pdf]

Table S7. Comparison of systemic and ophthalmological outcomes between infants treated with anti-vascular endothelial growth factor agents vs. infants treated with laser photocoagulation and/or cryotherapy for retinopathy of prematurity from January 2015 to December 2022 and for whom mother-infant linkage was available in the French National Health Data System (n = 174, sensitivity analysis 3)

**Outcome**	**Time period**	**Anti-VEGF agents (n = 111)**	**Laser photocoagulation and/or cryotherapy (n = 63)**	**OR (95% CI)***	***p***
Early neurodevelopmental disorders	Within the first 3 years of life**	59 (55.7%)	28 (44.4%)	1.4 (0.9 – 2.1)	0.13
Central neurological disorders	Within the first 3 years of life**	11 (10.4%)	8 (12.7%)	0.6 (0.3 – 1.2)	0.13
Cardiovascular diseases	Within the first 3 years of life**	11 (10.4%)	7 (11.1%)	0.7 (0.4 – 1.5)	0.39
All-cause mortality	Within the first year of life	5 (4.5%)	0 (0.0%)	NA	0.93
All-cause rehospitalization	Within the first year of life**	73 (68.9%)	48 (76.2%)	0.7 (0.4 – 1.1)	0.16
Surgery for retinal detachment or vitreous hemorrhage	Within the first year of life**	9 (8.5%)	7 (11.1%)	1.0 (0.5 – 2.2)	0.94

* Weighting on a propensity score accounting for pre-eclampsia, postnatal age at the time of ROP treatment and severe intraventricular hemorrhage.

** Analyses were performed on 106 infants treated with anti-VEGF and 63 infants treated with laser and cryotherapy after exclusion of infants who died before outcome assessment.

Abbreviations: Anti-VEGF, anti-Vascular Endothelial Growth Factor; NA, not applicable; ROP, retinopathy of prematurity.
