# Supplementary material for: Neurodevelopmental Disorders in Infants Treated with Anti-VEGF Agents versus Laser Photocoagulation or Cryotherapy for Retinopathy of Prematurity
Source: Ophthalmol Sci. 2026 Aug 17;6(11):101371. doi: 10.1016/j.xops.2026.101371 (PMC13654026; doi:10.1016/j.xops.2026.101371)
Supplement: Supplementary file 8 [file mmc8.pdf]

Table S8. Baseline characteristics of very preterm newborns treated for a retinopathy of prematurity from January 2015 to December 2019, for whom mother-infant linkage was available in the French National Health Data System before and after weighting (primary analysis population, sensitivity analysis 4)

	Before weighting			After weighting*		
	**Anti-VEGF agents (n = 59)**	**Laser photocoagulation and/or cryotherapy (n = 43)**	**ASD**	**Anti-VEGF agents**	**Laser photocoagulation and/or cryotherapy**	**ASD**
**Newborn characteristics**						
Gestational age at birth (weeks of amenorrhea)	25.6 ± 2.2	25.4 ± 1.7	0.09	25.5 ± 2.2	25.4 ± 1.6	0.03
Female	27 (45.8%)	18 (41.9%)	0.04	24 (43.9%)	14 (41.0%)	0.03
Birthweight, g	741 ± 294	720 ± 160	0.07	722 ± 274	711 ± 169	0.04
Postnatal age at the time of ROP treatment, days	47.0 ± 45.0	58.5 ± 50.8	0.25	48.0 ± 46.8	57.9 ± 49.7	0.22
Intrauterine hypoxia	13 (22.0%)	15 (34.9%)	0.13	13 (23.6%)	11 (30.2%)	0.07
Birth asphyxia	22 (37.3%)	17 (39.5%)	0.02	21 (38.2%)	13 (36.0%)	0.02
Respiratory distress of newborn	54 (91.5%)	40 (93.0%)	0.02	49 (90.7%)	33 (93.3%)	0.03
Ventilatory support	56 (94.9%)	42 (97.7%)	0.03	51 (94.0%)	34 (97.9%)	0.04
Infectious congenital diseases	11 (18,6%)	4 (9.3%)	0.09	11 (19.8%)	3 (7.2%)	0.13
Cardiovascular disorders originating in the perinatal period	27 (45.8%)	17 (39.5%)	0.06	24 (45.1%)	15 (43.7%)	0.01
Disturbances of cerebral status of newborn	4 (6.8%)	1 (2.3%)	0.04	3 (4.9%)	1 (3.8%)	0.01
Severe intraventricular hemorrhage	18 (30.5%)	6 (14.0%)	0.17	13 (23.2%)	8 (21.9%)	0.01
Necrotizing enterocolitis	8 (13.6%)	4 (9.3%)	0.04	7 (13.5%)	3 (7.2%)	0.06
Beneficiary of the French Complementary Health Solidarity	19 (32.2%)	15 (34.9%)	0.03	18 (33.2%)	12 (35.0%)	0.02
High social deprivation index (5th quintile) **	20 (40.8%)	11 (26.8%)	0.14	23 (42.4%)	10 (27.8%)	0.15
**Delivery characteristics**						

Caesarean section	38 (64.4%)	20 (47.6%)	0.17	32 (59.2%)	18 (52.2%)	0.07
Level 3 maternity hospital	50 (84.8%)	36 (83.7%)	0.01	48 (82.9%)	30 (84.4%)	0.02
Length of stay in maternity hospital, days	72.0 ± 50.6	71.1 ± 41.2	0.02	70.8 ± 50.3	72.3 ± 39.0	0.03
**Maternal characteristics**						
Maternal age at delivery, years	30.6 ± 6.5	30.2 ± 5.3	0.06	30.3 ± 6.4	30.4 ± 5.1	0.02
Preexisting hypertension before pregnancy	4 (6.8%)	1 (2.3%)	0.04	4 (6.9%)	2 (7.1%)	0.00
Preeclampsia	13 (22.0%)	2 (4.7%)	0.17	8 (14.6%)	5 (13.2%)	0.01
Gestational diabetes	8 (13.6%)	2 (4.7%)	0.09	8 (15.1%)	1 (3.8%)	0.11
Premature rupture of membranes	2 (3.4%)	3 (7.0%)	0.04	1 (2.5%)	2 (5.4%)	0.03
Placenta praevia	1 (1.7%)	3 (7.0%)	0.05	1 (2.1%)	2 (5.8%)	0.04
Retroplacental hematoma	7 (11.9%)	5 (11.6%)	0.00	6 (11.2%)	3 (9.4%)	0.02
Chorioamnionitis	11 (18.6%)	7 (16.3%)	0.02	11 (20.7%)	5 (14.3%)	0.06
Folic acid supplementation	21 (35.6%)	17 (39.5%)	0.04	19 (35.3%)	14 (40.5%)	0.05
Dispensing of psychotropic medications in the year prior to pregnancy	13 (22.0%)	8 (18.6%)	0.03	13 (23.2%)	6 (17.8%)	0.05

* Weighting on a propensity score accounting for preeclampsia, severe intraventricular hemorrhage, gestational age and birthweight.

** Missing data for n = 12 newborns

Abbreviations: Anti-VEGF, anti-vascular endothelial growth factor; ASD, absolute standardized difference.
