# Supplementary material for: Neurodevelopmental Disorders in Infants Treated with Anti-VEGF Agents versus Laser Photocoagulation or Cryotherapy for Retinopathy of Prematurity
Source: Ophthalmol Sci. 2026 Aug 17;6(11):101371. doi: 10.1016/j.xops.2026.101371 (PMC13654026; doi:10.1016/j.xops.2026.101371)
Supplement: Supplementary file 9 [file mmc9.pdf]

Table S9. Comparison of systemic and ophthalmological outcomes between infants treated with anti-vascular endothelial growth factor agents vs. infants treated with laser photocoagulation and/or cryotherapy for retinopathy of prematurity from January 2015 to December 2019, and for whom mother-infant linkage was available in the French National Health Data System (n = 102, primary analysis population, sensitivity analysis 4)

**Outcome**	**Time period**	**Anti-VEGF agents (n = 59)**	**Laser photocoagulation and/or cryotherapy (n = 43)**	**OR (95% CI)***	***p***
Early neurodevelopmental disorders**	Within the first 6 years of life	40 (71.4%)	25 (58.1%)	1.5 (0.8 – 2.7)	0.17
Central neurological disorders**	Within the first 6 years of life	6 (10.7%)	3 (7.0%)	1.5 (0.5 – 4.0)	0.46
Cardiovascular diseases**	Within the first 6 years of life	4 (7.1%)	5 (11.6%)	0.4 (0.2 – 1.1)	0.07
All-cause mortality	Within the first year of life	3 (5.1%)	0 (0.0%)	NA	0.92
All-cause rehospitalization**	Within the first year of life	33 (58.9%)	31 (72.1%)	0.5 (0.3 – 0.9)	0.03
Surgery for retinal detachment or vitreous hemorrhage**	Within the first year of life	6 (10.7%)	3 (7.0%)	1.1 (0.4 – 2.8)	0.82

* Weighting on a propensity score accounting for pre-eclampsia, severe intraventricular hemorrhage, gestational age and birthweight.

** Analyses were performed on 56 infants treated with anti-VEGF and 43 infants treated with laser and cryotherapy after exclusion of infants who died before outcome assessment.

Abbreviations: Anti-VEGF, anti-vascular endothelial growth factor; NA, not applicable.
